# Collagen as Coating Material for 45S5 Bioactive Glass-Based Scaffolds for Bone Tissue Engineering

**DOI:** 10.3390/ijms19061807

**Published:** 2018-06-19

**Authors:** Jasmin Hum, Aldo R. Boccaccini

**Affiliations:** Institute of Biomaterials, Department of Materials Science and Engineering, University of Erlangen-Nuremberg, Cauerstrasse 6, 91058 Erlangen, Germany

**Keywords:** bioactive glass, collagen, scaffolds, bone tissue engineering, surface functionalization

## Abstract

Highly porous 45S5 bioactive glass-based scaffolds were fabricated by the foam replica technique and coated with collagen by a novel method. After an initial cleaning step of the bioactive glass surface to expose reactive –OH groups, samples were surface functionalized by (3-aminopropyl)triethoxysilane (APTS). Functionalized scaffolds were immersed in a collagen solution, left for gelling at 37 °C, and dried at room temperature. The collagen coating was further stabilized by crosslinking with 1-ethyl-3-(3-dimethylaminopropyl)carbodiimide (EDC) and *N*-hydroxysuccinimide (NHS). Applying this coating method, a layer thickness of a few micrometers was obtained without affecting the overall scaffold macroporosity. In addition, values of compressive strength were enhanced by a factor of five, increasing from 0.04 ± 0.02 MPa for uncoated scaffolds to 0.18 ± 0.03 MPa for crosslinked collagen-coated scaffolds. The composite material developed in this study exhibited positive cell (MG-63) viability as well as suitable cell attachment and proliferation on the surface. The combination of bioactivity, mechanical competence, and cellular response makes this novel scaffold system attractive for bone tissue engineering.

## 1. Introduction

The interdisciplinary field of tissue engineering combines approaches from biology, engineering, and material science to develop alloplastic grafts or scaffolds for substitution or repair of tissue damaged by disease or trauma [1,2]. Especially in bone tissue engineering, there is an important demand for scaffolds as the availability of autografts, the current gold standard for bone transplants [3,4], is strongly limited. For the fabrication of a nonbiological matrix, which acts as a temporary scaffold structure to support cell attachment, growth, and proliferation [5,6], different biomaterials are available. The scaffold structure should offer suitable mechanical properties, adequate pore size, and a high degree of porosity to enable tissue ingrowth and vascularization, whereas the applied biomaterial should also possess osteoconductivity, osteoinductivity, and should be biodegradable [7]. In this context, bioactive glasses (BGs) are attracting increasing attention based on their high bioactivity, osteogenic potential, biodegradability, and angiogenic effects [8,9]. However, due to the brittle nature of glasses, scaffolds produced from these materials are usually not suitable for load-bearing applications. A common strategy to improve the mechanical performance of such scaffolds is the development of composite materials. By reinforcement with a polymer phase, the mechanical competence can be improved [10]. Collagen is the most abundant protein in the extracellular matrix, being also a very popular biomaterial [11]. Due to its natural origin, collagen offers suitable binding sites for cellular attachment, being thus a very interesting bone substitute material, usually in combination with bioactive, inorganic phases [12].

Collagen is a very versatile biomaterial and can be processed in different ways to form sheets, sponges, or injectable scaffolds, among others [13,14]. There is a broad range of applications of collagen in the biomedical field. Collagen films are used, for example, as graft material for corneal replacement or for treatment of infections when loaded with anti-inflammatory drugs [14,15]. Furthermore, collagen can be combined with bone morphogenetic proteins (BMPs), which can be used to stimulate osteoinduction [16]. For wound dressing, three-dimensional collagen sponges are often applied as they can absorb exudates and offer a natural barrier for bacterial infections [11]. Collagen is also applied as a coating material [17,18]. For example, Douglas and Haugen [19] described the coating of polyurethane scaffolds with collagen to improve the cell response. In most cases, collagen applied as a coating material aims to increase the biological activity. However, the mechanical improvement that can be achieved by collagen coatings, based on the high tensile strength of collagen fibers, is not usually investigated.

In this study, bioactive glass-based scaffolds were produced by the foam replica technique [20] and coated by collagen. The approach of using collagen as a coating material for BG-based scaffolds has been put forward before [21]. However, the previously reported method [21] usually leads to extremely thin coatings as only a collagen monolayer can attach on the scaffold surface. Therefore, a new coating method is introduced in this work, which enables application of a homogenous collagen layer of a few microns thickness on the surface of BG-based scaffolds. It is expected that such novel collagen coating will enhance the biologically activity of the scaffolds, maintaining a suitable mechanical competence for bone tissue engineering applications.

Samples were analyzed in terms of bioactivity, release behavior, and mechanical performance. In addition, biocompatibility was studied in vitro using a human osteosarcoma cell line (MG-63).

## 2. Results

### 2.1. Morphological and Microstructural Characterization

**Figure 1:**
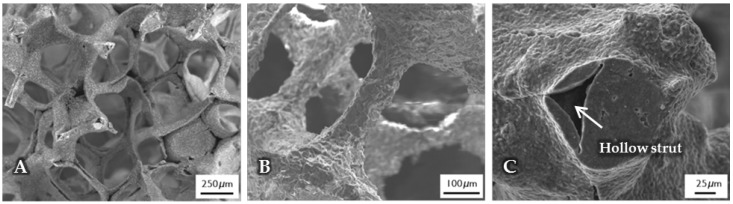
SEM images of as-fabricated bioactive glass-based scaffolds after sintering, at lower (**A**) and higher (**B**) magnifications. The hollow nature of the struts can be clearly seen (**C**) and can be attributed to the burn-out of the sacrificial polyurethane (PU) foam.

The micro- and macrostructure of as-fabricated BG-based scaffolds was investigated by SEM and can be seen in Figure 1. The images show the typical structure of scaffolds fabricated by the foam replica technique. Samples exhibit a highly interconnected porous structure similar to cancellous bone (Figure 1A). As the sacrificial polyurethane (PU) foam is burned out during sintering, the struts exhibit a hollow nature (Figure 1C). Pore size ranges between 250 and 500 µm, whereas struts show diameters from 50 to 100 µm (Figure 1B).

**Figure 2:**
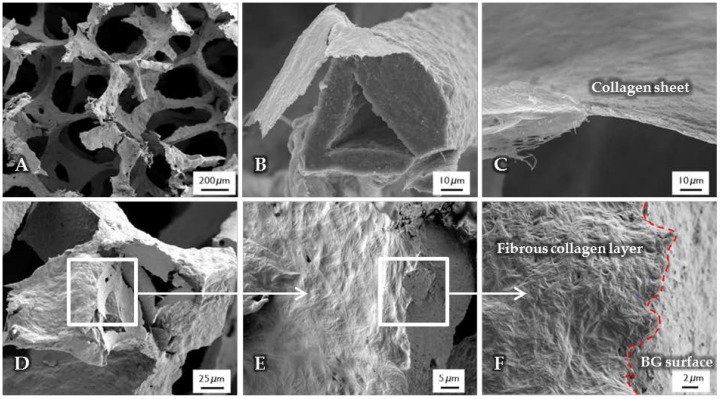
SEM images of uncrosslinked, collagen-coated bioactive glass-based scaffolds. The fibrous collagen layer can be clearly seen (**B**). After the coating process, the overall macroporosity of the scaffold is not affected (**A**). The collagen layer exhibits a thickness of a few micrometers (**C**). At the interface ((**D**–**F**), different magnifications), the rough bioactive glass surface can be clearly distinguished from the fibrous collagen layer.

After the surface functionalization of the BG-based scaffolds, a collagen coating was applied. The structure, observed by SEM, is shown in Figure 2. Due to the high shrinkage rate during the drying process of the collagen gel, collagen fibers are contracted and orientated along the structure of the scaffold, which results in a dense and homogenous layer wrapped around the struts (Figure 2B), while the macroporosity of the structure is not affected (Figure 2A). Cross-section images show a layer thickness of a few micrometers (Figure 2C). At the interface (Figure 2D–F), the difference between the fibrous collagen structure and the rough bioactive glass surface can be clearly observed. In addition, by the presence of the coating, the handling of the scaffolds was improved, which is also demonstrated by their higher compressive strength in comparison to uncoated scaffolds (see Section 2.6).

### 2.2. Surface Analysis

**Figure 3:**
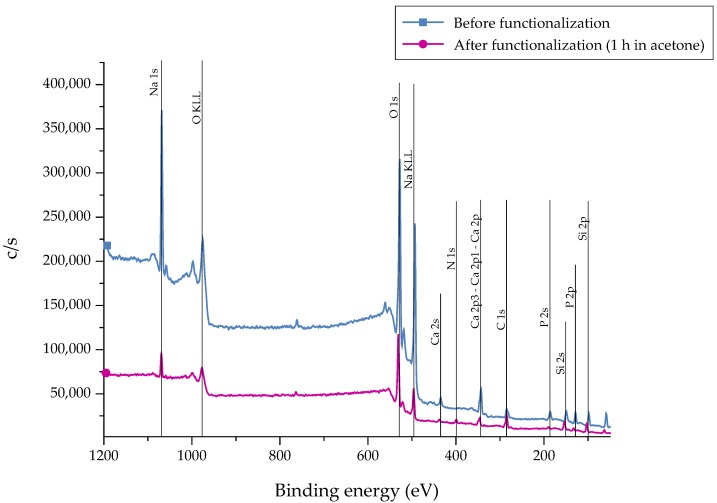
XPS spectra of 45S5 bioactive glass (BG) surfaces before and after the silanization process in acetone +2 vol % APTS for 1 h.

After the silanization process of the bioactive glass surface, the presence of APTS molecules was detected by X-ray Photoelectron Spectroscopy (XPS) measurements. Different markers can be applied. As carbon can represent a source of contamination, nitrogen (N) was chosen as representative marker which is not part of the 45S5 bioactive glass substrate [22]. As a reference, as-received samples were chosen. The recorded spectra are presented in Figure 3. The associated atomic concentration of each element is shown in Table 1. A noticeable change in the spectra can be seen after functionalization as the intensity of the peaks is reduced after 1 h of immersion, indicating the fast dissolution process of the bioactive glass substrate. This fact is supported by the decreased atomic concentration of bioactive glass-related elements such as calcium, sodium, and phosphorous (Table 1). Due to the reaction of APTS molecules with free –OH groups on the surface, the peak at 533 eV (O1s) decreases after functionalization. In addition, the peak at 400 eV (N1s), which appears in return, confirms the presence of N-containing groups, attributed to the successful coupling of APTS molecules to the surface. Also, an increased atomic concentration of nitrogen and silicon can be found. 

### 2.3. Crosslinking Process

**Figure 4:**
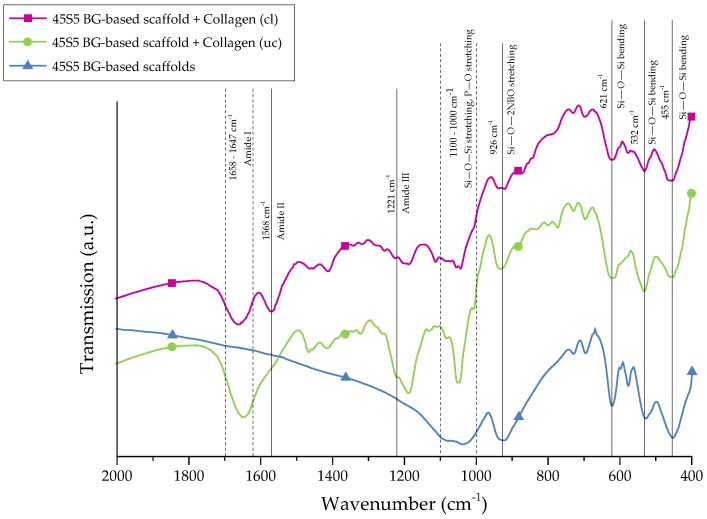
FTIR spectra of 45S5 BG-based scaffolds and collagen-coated 45S5 BG-based scaffolds before and after crosslinking. Relevant peaks are discussed in the text.

For further stabilization of the collagen coating, chemical crosslinks were introduced by treatment with EDC ((*N*-3-Dimethylaminopropyl)-*N*′ethylcarbodiimide) and NHS (*N*-Hydroxysuccinimide). The success of crosslinking was evaluated by FTIR. Figure 4 shows the FTIR spectra of collagen-coated bioactive glass-based scaffolds with and without crosslinking. For comparison, results of as-fabricated scaffolds are also shown and the most relevant peaks are labeled.

Uncoated 45S5 bioactive glass-based scaffolds show typical peaks at 455 cm^−1^ (Si–O–Si bending) and in the region between 1100 and 1000 cm^−1^ (Si–O–Si stretching), which can be attributed to the Si–O–Si vibrational modes in the glass network [23,24]. Due to the overlap of Si–O–Si stretching and P–O stretching in the area between 1100 and 1000 cm^−1^, these bands are difficult to distinguish [25]. Sharp peaks at 455, 532, and 621 cm^−1^ indicate a high crystallinity of the bioactive glass-based samples attributed to the Si–O–Si vibrational mode of the developed crystal phase [26]. The vibrational mode of the Si–O–2NBO (nonbridging oxygen) bond is visible by the peak at 926 cm^−1^, resulting from the formation of SiO^−^ groups in the glass network [23,25,27]. After the collagen coating, the formation of new peaks in the area between 1700 and 1200 cm^−1^ confirms the presence of the protein on the surface. These peaks can be attributed to amide I [25,28,29] (C=O stretching), typical for the triple helical structure in nondenatured collagen, and amide III [30] (N–H deformation). The additional amide-type bond (amide II) at 1568 cm^−1^, which is present in the spectrum of crosslinked samples, confirms the success of the crosslinking process [28,30].

**Figure 5:**
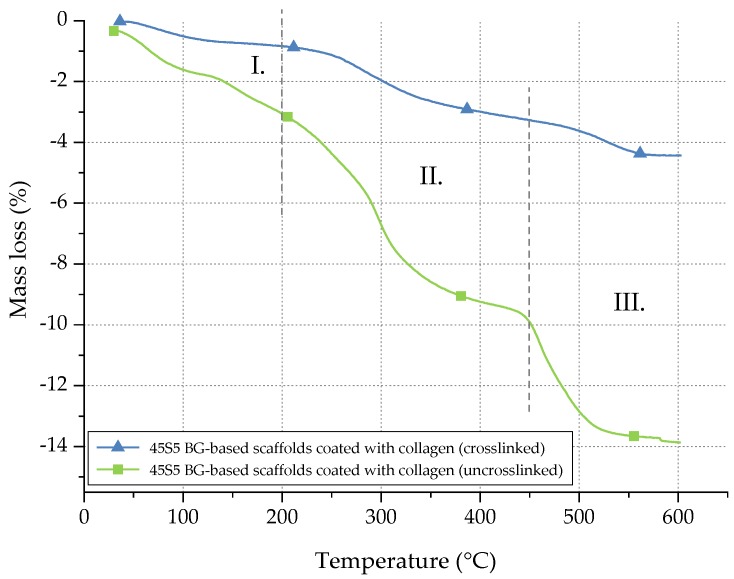
Mass loss of uncrosslinked and crosslinked collagen on 45S5 BG-based scaffolds during TGA.

In order to quantify the amount of collagen before and after crosslinking, thermogravimetric measurements were carried out. Temperature and weight loss were continuously recorded and are presented in Figure 5.

The mass loss of collagen can be divided into three different stages [31]. Between room temperature (RT) and 200 °C (stage I), the mass loss can be attributed to the evaporation of water. In stage II (200–450 °C), collagen molecules are decomposed. In the last step (stage III), between 450 and 600 °C, residual organic components of the collagen coating are pyrolysed. Considering the mass loss in stage II, the collagen amount of uncrosslinked samples can be determined as 6.5 wt %, whereas only 2 wt % of collagen is left after crosslinking. The difference can be explained by the release of uncrosslinked collagen during the process of crosslinking, whereas only collagen fibrils, fixed in the crosslinked network, remain. Indeed, during the crosslinking process, the collagen fibrils are stabilized. However, the crosslinking process itself takes a few hours. During this time, some collagen fibrils may be released and are not fixed in the network. Thus, a given amount of collagen is lost during the crosslinking process, resulting in a lower amount of collagen after crosslinking.

### 2.4. Evaluation of Bioactivity

**Figure 6:**
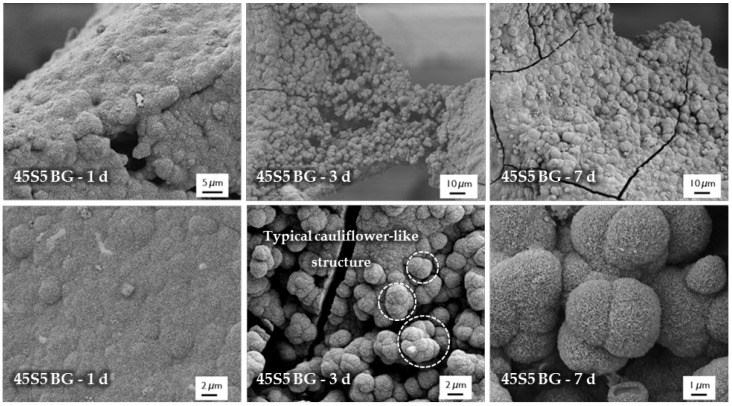
SEM images of as-fabricated 45S5 BG-based samples after immersion in simulated body fluid (SBF) for 1, 3, and 7 days (at different magnifications).

**Figure 7:**
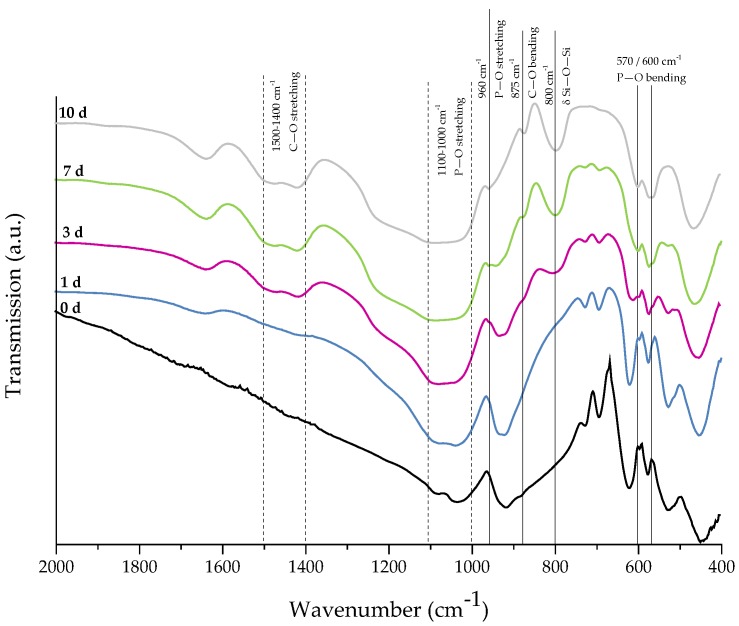
FTIR spectra of as-fabricated 45S5 BG-based samples after 0, 1, 3, 7, and 10 days of immersion in SBF. Relevant peaks are discussed in the text.

For evaluation of the bone-bonding ability of uncoated and collagen-coated scaffolds, the bioactive behavior was investigated by immersion in simulated body fluid (SBF). The formation of hydroxyapatite on the surface of different samples was observed by SEM and confirmed by FTIR measurements. Figure 6 shows SEM images of as-fabricated bioactive glass-based scaffolds after immersion in SBF for 1, 3, and 7 days. After one day, calcium phosphate precipitates are already visible. The typical morphology of hydroxyapatite (HA) can be clearly recognized after three days of immersion in SBF, qualitatively assessed by the cauliflower-like shape [32]. After seven days, the entire surface is covered by a dense layer of HA. Also, the FTIR spectra, which can be seen in Figure 7, confirm the formation of hydroxyapatite on the surface of bioactive glass-based scaffolds during immersion in SBF. The growing double peak at 600 and 570 cm^−1^ can be attributed to the P–O bending vibrations related to the PO_4_^3−^ group in the crystalline layer of HA [33]. Also, the growing peak in the area between 1100 and 1000 cm^−1^ and the small shoulder at 960 cm^−1^ can be related to the phosphate group (P–O stretching) [33,34]. Developed peaks at 875 cm^−1^ and in the region between 1500 and 1400 cm^−1^ can be attributed to the C–O bending and stretching, respectively, and belong to the CO_3_^2−^ groups in the carbonated HA layer [33,35]. Due to the formation of a silica-rich layer, a peak at 800 cm^−1^ can be observed [27].

**Figure 8:**
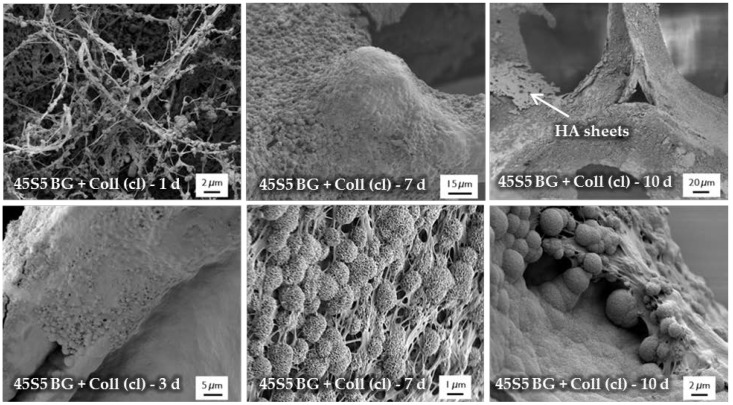
SEM images of collagen (Coll)-coated 45S5 BG-based scaffolds (cl) after immersion in SBF for 1, 3, 7, and 10 days (at different magnifications). The formation of a mineralized collagen layer can be observed.

**Figure 9:**
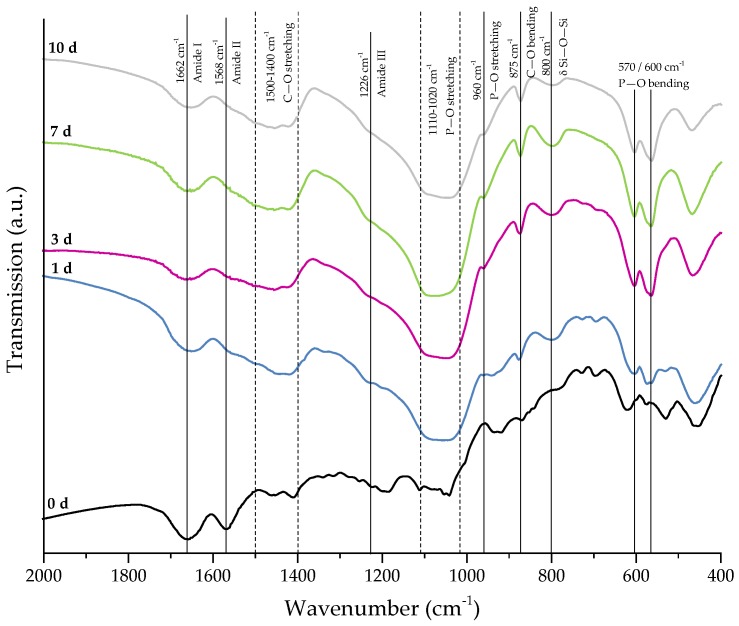
FTIR spectra of collagen-coated 45S5 BG-based scaffolds (crosslinked) after 0, 1, 3, 7, and 10 days of immersion in SBF. Relevant peaks are discussed in the text.

Figure 8 shows SEM images demonstrating the bioactive behavior of 45S5 bioactive glass-based scaffolds coated by collagen (crosslinked). The formation of small hydroxyapatite nodules along the collagen fibers can be observed after one day. With longer immersion time in SBF, HA microcrystals are directly deposited on the fibrous structure and remain well-embedded in the collagen matrix. After 10 days, the layer of collagen is mostly mineralized. The literature describes the bioactive behavior of collagen as being due to the existence of nucleation sites in the collagen network. Nucleation sites such as –COOH groups interact with the Ca^2+^ ions in the SBF solution and support the formation of hydroxyapatite [36,37,38]. This interaction can be also seen in the FTIR spectra (Figure 9), due to the shifted peak of amide I and the disappearance of amide II and III peaks after immersion in SBF. Characteristic peaks, indicating the formation of HA on the surface, are developed in the wavenumber region between 1100 and 1000 cm^−1^ and the small shoulder at 960 cm^−1^, and at 875, 800, 600, and 570 cm^−1^, as explained above. It should be mentioned that the amount of HA formed on the different scaffold types (shown in Figure 6 and Figure 8) was not quantified in this study, so the results remain qualitative. Nevertheless, summarizing the results, it can be stated that the bioactive behavior of 45S5 bioactive glass-based scaffolds is not compromised by the presence of collagen, but it is even enhanced due to the presence of nucleation sites in the collagen structure.

### 2.5. Release Behavior

To investigate the stability of the collagen coating on the surface of the bioactive glass-based scaffolds and to evaluate the delivery capability, release studies were carried out in different media. An overview of the investigated samples is given in Table 2. Collagen release was determined depending on the cleaning and functionalization process.

**Figure 10:**
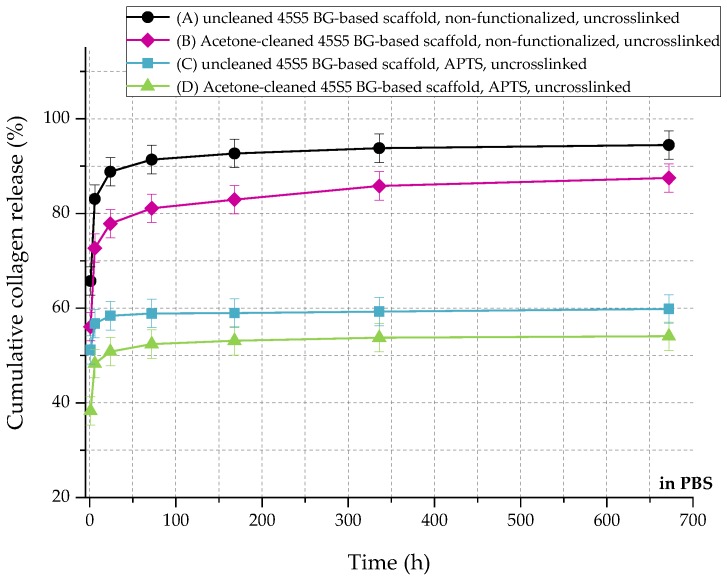
Cumulative collagen release from different types of 45S5 BG-based scaffolds in PBS.

In relation to the scaffolds listed in Table 2, Figure 10 shows the cumulative amount of released collagen in phosphate buffered saline (PBS) up to 28 days. All curves show an initial burst release followed by a plateau. During the process of collagen coating, a collagen mesh of single collagen fibers is wrapped around the struts of the 45S5 bioactive glass-based scaffold (Figure 2). In the case of nonfunctionalized samples (A and B), the collagen is only physically adsorbed on the surface due to missing binding sites. Because of missing crosslinks, water molecules penetrate the collagen mesh very fast, leading to swelling of the fibrous structure. Macromolecules are released over time and diffuse, which is shown by a high release rate of around 90% in the first 24 h. After 28 days, less than 10% of collagen is left on the surface of the scaffolds. By introducing a silanized surface (C and D), collagen can bind covalently to the bioactive glass surface, which results in a reduced release rate. Similar results were also reported before [21]. In addition, results show that the amount of covalently bonded collagen could be increased by introducing an initial cleaning step, due to more reactive –OH groups on the surface. However, initial burst release up to 50% can be observed as the functionalization process only affects collagen close to the surface. Collagen molecules, which are not covalently bonded to the surface, still diffuse and are released very fast. 

**Figure 11:**
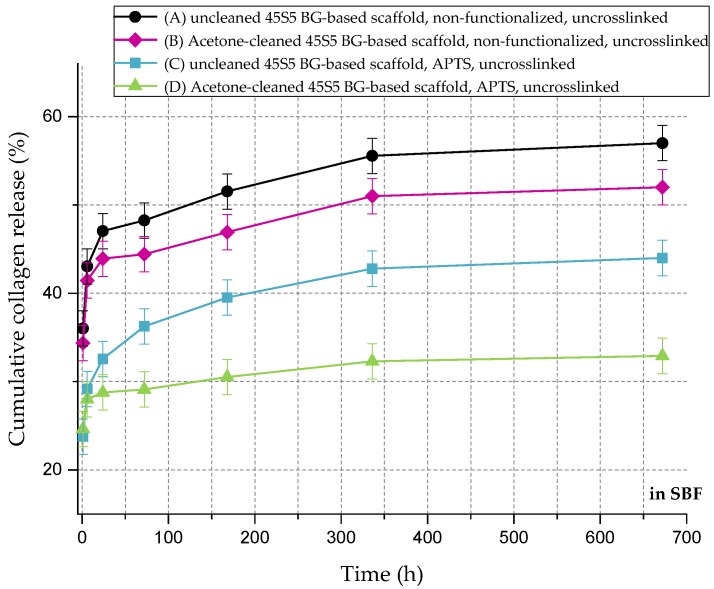
Cumulative collagen release from different types of 45S5 BG-based scaffolds in SBF.

Figure 11 shows the cumulative collagen release in SBF for up to 28 days. The collagen release kinetics is similar to that in PBS, although the overall amount is remarkably lower. Even without surface functionalization, max 60% of the initial collagen is released. As the collagen is mineralized due to the immersion in SBF (Figure 8), nodules of hydroxyapatite are embedded in the collagen matrix, which could explain the reduced release rate of the protein. 

**Figure 12:**
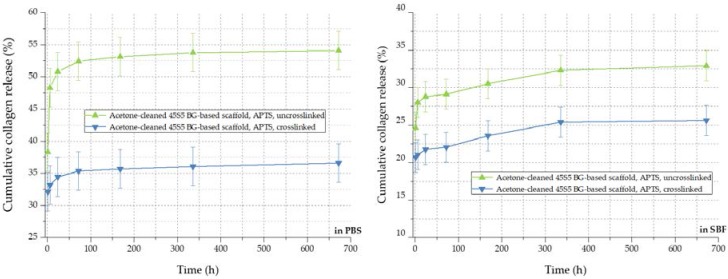
Collagen release kinetics of collagen-coated 45S5 BG-based scaffolds in PBS (**left**) and SBF (**right**) before and after crosslinking.

By the crosslinking process, the cumulative release rate of collagen can be further decreased, as visible in Figure 12. The amount of released collagen could be further reduced from 54% to 36% in PBS and from 32% to 26% in SBF, respectively. In addition to covalently bonded collagen molecules on the bioactive glass-based surface, the crosslinked network of collagen exhibits a lower release rate during the 28 days of immersion. In conclusion, the collagen release from 45S5 bioactive glass-based scaffolds can be adapted introducing a silanized surface and by varying the degree of crosslinks, depending on the application, thus providing a very versatile system.

### 2.6. Mechanical Characterization

**Figure 13:**
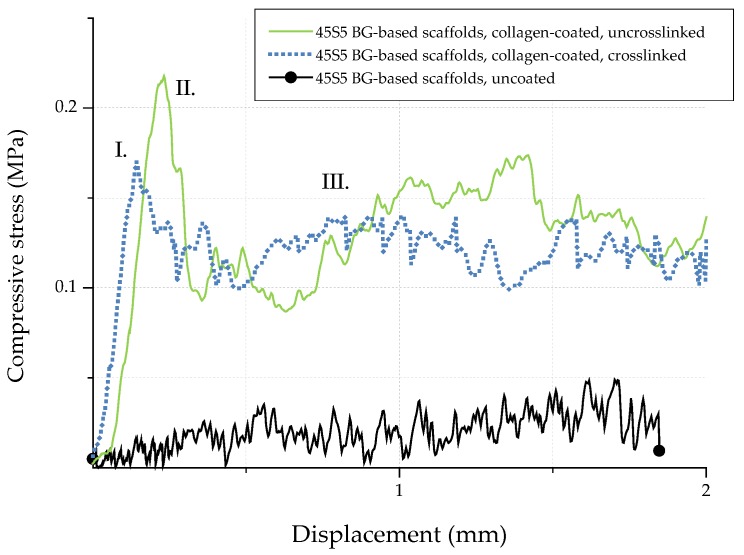
Exemplary stress-displacement curves for 45S5 BG-based scaffolds with and without collagen coating.

In order to investigate the potential of collagen-coated bioactive glass-based scaffolds for application in bone tissue engineering, the compressive strength (σ) of uncoated and collagen-coated samples was determined. Figure 13 shows exemplary stress-displacement curves for uncoated and collagen-coated samples, which can be divided into three different regions [20]. The compressive stress increases constantly in region I until a maximum stress is reached and the scaffolds’ struts fail. As a result, the curve shows a negative slope in region II. Due to progressive compression in region III, the broken struts are densified and the compressive stress increases. As the struts exhibit a hollow nature, uncoated bioactive glass-based scaffolds show low compressive strength values (0.04 ± 0.02 MPa). The shape of the stress-displacement curve and such jagged characteristics are common in this type of scaffolds tested in compression [10,20,21,22]. Moreover, the compressive strength values for uncoated bioactive glass-based scaffolds are comparable with results found in the literature [39,40,41] and are typical for scaffolds produced by the foam replica technique. However, Bellucci et al. [42] describe compressive strength values of up to 0.8 MPa for similar bioactive scaffolds (based on a modified replication method), which can be attributed to the reduced porosity of the samples.

Due to the collagen coating, microcracks present on the surface of the scaffolds are filled by collagen, resulting in enhanced mechanical strength. The effect of polymer infiltration and the associated reinforcement is well-known and has been described elsewhere [10]. Similar values of compressive strength of 0.21 ± 0.03 MPa and 0.18 ± 0.03 MPa were achieved for uncrosslinked and crosslinked collagen-coated scaffolds, respectively. In order to simulate the environment in vitro, samples were additionally soaked in PBS. After 1 h, stress-displacement curves were recorded and compared with those of dry samples. It was observed that due to the rehydration of the coating, collagen transforms into a hydrogel, which results in a drop of the compressive strength (to values of <0.1 MPa). However, even if the compressive strength is reduced after the scaffold comes into contact with an aqueous medium, the biomedical application of collagen-coated BG-based scaffolds in vivo is not compromised, since due to the collagen coating, the scaffolds can be safely handled and processed.

### 2.7. Cell Studies

#### 2.7.1. Cell Viability and Relative Proliferation

**Figure 14:**
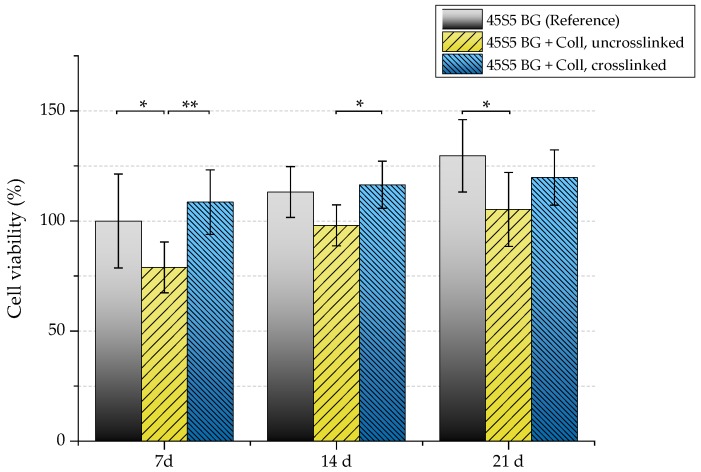
Cell viability of MG-63 cells of different scaffold types (absorbance at 450 nm) after 7, 14, and 21 days. Significance levels: * *p* < 0.05, ** *p* < 0.01 (Bonferroni’s post-hoc test was used).

The biocompatibility of scaffolds was evaluated by investigating their interaction with osteoblast-like cells (MG-63). Uncoated and collagen-coated samples (with and without crosslinks) were seeded with MG-63 cells and cultivated in static conditions for 7, 14, and 21 days. Figure 14 shows the viability of seeded MG-63 cells. After 21 days, uncrosslinked collagen-coated samples exhibit significant lower cell viability (105%) compared to as-fabricated scaffolds without coating (130%). Because uncrosslinked collagen is released very fast (see Section 2.5), a high cell number is lost at the beginning of cultivation as cells directly attach to the collagen matrix. This is already visible after seven days, where a noticeable difference in the cell viability of samples coated with uncrosslinked collagen can be seen compared to the uncoated reference. Therefore, higher cell viability can be detected for crosslinked samples.

**Figure 15:**
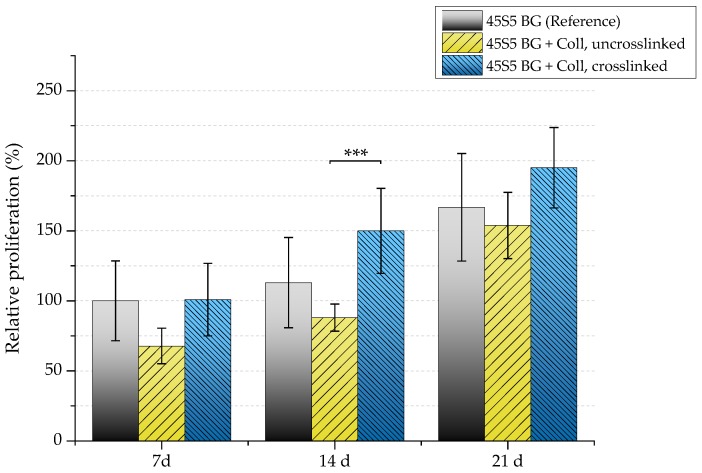
Relative proliferation of MG-63 cells on different scaffold types (absorbance at 450 nm) after 7, 14, and 21 days. Significance level: *** *p* < 0.001 (Bonferroni’s post-hoc test was used).

Because a relatively high cell number is lost in uncrosslinked collagen-coated samples, a reduced proliferation rate can also be detected in this case (Figure 15). After seven days, samples showed a relative proliferation below 75%. A significant difference can be seen after 14 days comparing uncrosslinked and crosslinked collagen-coated scaffolds. However, remaining cells were found to be able to recover, which results in a similar proliferation rate compared to pure 45S5 bioactive glass-based scaffolds after 21 days of incubation.

#### 2.7.2. Cell Morphology

**Figure 16:**
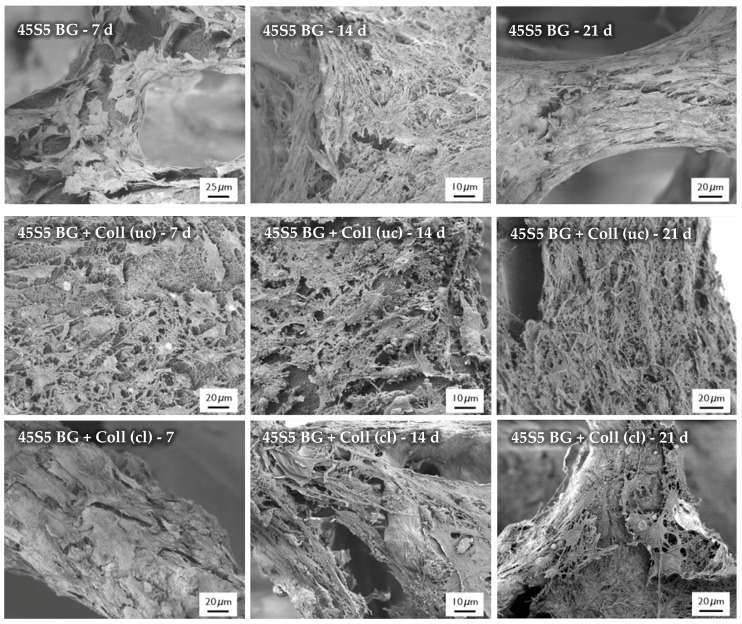
SEM images of seeded MG-63 cells on different types of scaffolds for 7, 14, and 21 days (at different magnifications).

After the cultivation of scaffolds with MG-63 cells, the morphology of cells was observed by SEM, as shown in Figure 16. SEM images show the cell morphology after different time points and confirm the results which were achieved by the evaluation of cell viability and relative proliferation. Cells with their typical spindle-shaped morphology can be found on the surface of every sample, indicating the high biocompatibility of the applied collagen coating. After 21 days of incubation, even multilayer growth of MG-63 cells can be observed. Due to the homogenous distribution of cells, it is assumed that scaffolds exhibit suitable pore size and porosity, which enables the infiltration of cells. However, a denser layer of cells can be found on the outer layer of the scaffolds, attributed to the gradient of nutrient and oxygen supply related to the static cultivation system.

## 3. Discussion

Due to its highly bioactive character, 45S5 bioactive glass shows great potential to be used as bone-substitute material. However, scaffolds produced from this material are highly brittle and the relative low fracture strength of 45S5 bioactive glass-based scaffolds is a significant drawback for their application in bone tissue engineering, particularly in load-bearing sites. Even the handling of such highly brittle scaffolds is very challenging. On the other hand, polymers are in general flexible and provide chemical versatility, thus the formation of bioactive glass–polymer composites is being explored to provide bioactive and mechanically sound scaffolds [2].

For the reinforcement of highly porous glass or ceramic structures and to overcome their lack of mechanical stability, scaffolds can be toughened by a polymer coating, as reviewed by Philippart et al. [10] and Yunos et al. [2]. The related reinforcement can be attributed to the filling and bridging of microcracks present on the scaffold surface by a polymeric phase. The literature mainly describes synthetic polymers, for example, polycaprolactone (PCL) [43,44] or poly (d,l-lactic acid) (PDLLA) [45,46], as the coating material. For example, to improve the compressive strength of bioactive glass-based scaffolds fabricated by the foam replica technique, Chen and Boccaccini [45] used PDLLA as the coating material. By dip-coating, the mechanical performance of the brittle samples could be considerably increased.

Natural polymers are an attractive alternative to synthetic polymers and are drawing attention as coating materials for scaffolds, taking into account that synthetic polymers can comprise negative byproducts when degrading. Natural polymers such as gelatin, silk, or collagen are often used as biomaterials and offer a wide range of properties, for example, biocompatibility, degradability, and nontoxicity. In addition, special sequences of amino acids (RGD) present in such biopolymers can improve cell attachment. For example, Metze et al. [41] used gelatin, a close relative of collagen, as a coating for 45S5 bioactive glass-based scaffolds to improve the compressive strength. Li et al. [47] showed increased mechanical performance as well as enhanced osteogenic differentiation behavior when coating ceramic scaffolds with silk.

In the present study, collagen was the polymer of choice due to its high biocompatibility and bioactivity. The collagen coating was applied by a combined process of surface functionalization and collagen immersion, leading to homogenous and thick collagen layers on the surface of BG-based scaffolds, while the overall porosity was not significantly hampered. By filling and bridging microcracks on the surface of the struts, which are caused by the sintering process during scaffold fabrication, the compressive strength of the scaffolds increased from 0.04 ± 0.02 MPa to 0.18 ± 0.02 MPa for uncoated and collagen-coated scaffolds, respectively. These values are at the lower boundary of the compressive strength of natural bone, which is reported to be between 0.2 and 4 MPa [20], and thus the collagen-coated scaffolds become attractive for application in bone tissue engineering. In addition, it is assumed that ingrowth of tissue in vivo will provide additional mechanical support, as reported in the literature [48].

Even though collagen transforms into a hydrogel in wet conditions, which could imply a loss of mechanical strength in vivo, the biomedical application of collagen-coated BG-based scaffolds is not negatively affected, as the in situ degradation of the collagen coating will occur in conjunction with the formation of hydroxyapatite on the surface of the scaffolds, due to the high bioactivity of the bioactive glass, providing local support to the structure. Indeed, when using scaffolds as bone-substituting materials, one important requirement is to ensure a safe handling of the alloplastic graft before implantation, for which the presence of the collagen coating is required. Due to the collagen coating, the otherwise brittle bioactive glass-based scaffolds can be safely handled and processed, and such a tough and stable matrix is suitable to fill bone defects during operation. The degradation of the collagen coating and the bioactive glass matrix starts immediately after implantation. Release products of collagen should enhance tissue regeneration and angiogenesis [49,50,51], while the ionic dissolution products of bioactive glass will further stimulate osteogenesis and angiogenesis by the enhanced expression of genes in cells, leading to bone formation and vascularization [52]. In addition, collagen offers many nucleation sites for the formation of hydroxyapatite, which is an important parameter for the interaction of a material with bone tissue, enhancing the intrinsic bioactivity of the bioactive glass scaffold. Thus, composite scaffolds that result from combining 45S5 bioactive glass and collagen offer high biocompatibility and bioactivity coupled with adequate structural integrity for safe handling of the scaffolds. In addition, collagen as a coating material not only influences cell attachment and proliferation, but it also offers a platform for the incorporation of biomolecules and drugs. By introducing a functionalized surface, combined with a crosslinked network of collagen molecules, the release rate of collagen (and incorporated drugs) can be easily controlled and applied as a drug delivery vehicle.

Overall, this study has introduced an alternative approach for the future development of composite scaffolds based on collagen and bioactive glasses. Even if significant challenges remain to enable the translation of the present scaffolds to clinical practice, the present results provide a well-founded basis for considering the newly developed scaffolds as a valid alternative for further investigations in this field.

## 4. Materials and Methods

### 4.1. Scaffolds

#### 4.1.1. Scaffold Production

Cylindrical and highly porous scaffolds (diameter = 0.75 mm, height = 0.4 mm) were produced by the foam replica process, originally described by Chen et al. [20]. Briefly summarized, sacrificial polyurethane (PU) foams (45 pores per inch (ppi), PL Bulpren S28133, Eurofoam Deutschland GmbH, Germany) were dip-coated in a BG-based slurry. For the preparation of the slurry, 0.3 wt % of binder (polyvinyl alcohol (PVA), fully hydrolyzed, M_w_ ~ 30,000, Merck Millipore, Darmstadt, Germany) was dissolved at 80 °C in ultrapure water (UPW) under stirring conditions. After cooling down to room temperature (RT), 2 wt % of disperser (KV 9062, Zschimmer & Schwarz GmbH & Co. KG, Lahnstein, Germany) and 50 wt % of BG powder were added. For the production of scaffolds, a commercially available melt-derived bioactive glass powder of the 45S5 composition (45S5 BG, in wt %: 45 SiO_2_, 24.5 CaO, 24.5 Na_2_O, and 6 P_2_O_5_) with particle size of ~2 µm was used (Vitryxx^®^, Schott, Mainz, Germany). After mixing the slurry homogenously, precut PU foams were immersed into the slurry, retrieved after 5 min, and squeezed to remove excess slurry. After drying at 60 °C for 2 h, the coating procedure was repeated. The samples were dried again at 60 °C and the received green bodies were finally sintered at 1050 °C to densify the structure.

#### 4.1.2. Surface Functionalization

**Figure 17:**
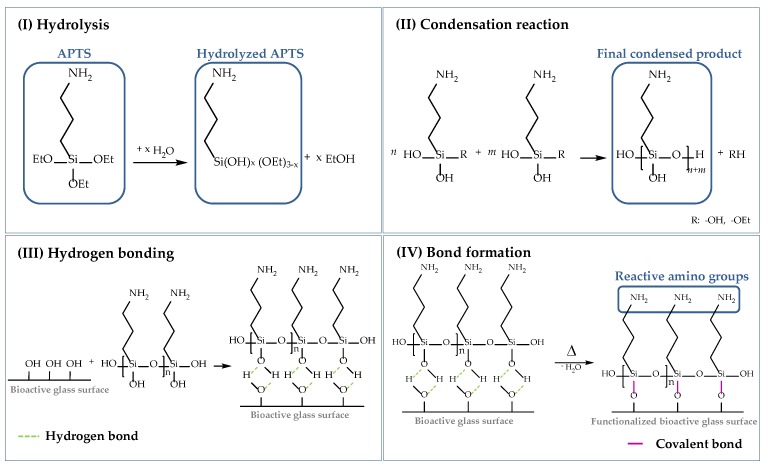
Surface functionalization of 45S5 bioactive glass-based scaffolds with APTS divided into (**I**) hydrolysis, (**II**) condensation reaction, (**III**) hydrogen bonding, and (**IV**) bond formation [50,54,55,56].

As already mentioned, cells attach to material surfaces in contact with proteins. Proteins reveal chemical complexity and a fragile nature and can change their native conformation considerably [21,53] due to electrostatic and hydrophobic interactions. To prevent the alteration of the biological functionality, proteins (in this case, collagen) can be grafted to the scaffold surface by chemical bonding. In this study, the surface of the bioactive glass-based scaffolds was functionalized using (3-aminopropyl)triethoxysilane (APTS), which is one of the most studied coupling agents and has been used in several studies before [21,54,55]. The following scheme (Figure 17) describes the surface functionalization of 45S5 BG-based scaffolds using APTS. The silanization process can be divided into four stages, namely: (I) hydrolysis, (II) condensation reaction, (III) hydrogen bonding, and (IV) bond formation.

To start the process of hydrolysis, adsorbed moisture on the surface is sufficient. Water molecules attack the ester bonds in the ethoxy groups (Si–OEt), which are hydrolyzed and replaced by hydroxyl groups (–OH) to form reactive silanol groups (Si–OH). Through a self-condensation process, silanol groups react with each other in step (II) to form stable siloxane bonds (Si–O–Si). The final condensed product can now react with –OH groups located on the BG surface. By hydrogen bonding, a network of siloxanes assembles on the surface of 45S5 BG-based scaffolds. As these bonds are not very stable, water is removed by a heat treatment and hydrogen bonds are replaced by covalent bonds in step (IV). To ensure a uniform deposition of reactive amino groups on the surface of bioactive glass-based scaffolds, an initial cleaning step was performed to remove contaminations and to activate reactive hydroxyl groups on the surface [56]. Based on previous work [56], an initial cleaning step was introduced before the surface of the samples was functionalized. Therefore, bioactive glass-based scaffolds were gently swiveled in acetone for 5 min, followed by rinsing in UPW. This process was repeated three times. Afterwards, samples were dried at 60 °C.

For surface functionalization, cleaned samples were immersed in a solution with 2 vol % APTS (99%, Sigma-Aldrich, Schnelldorf, Germany) in acetone (Acetone Technical, VWR Chemicals, Ismaning, Germany). After 1 h, scaffolds were removed, rinsed two times in UPW, and heat-treated at 100 °C for 2 h.

#### 4.1.3. Collagen Coating

Surface-functionalized bioactive glass-based scaffolds were coated with collagen using the following protocol. Solution A was prepared by mixing 1 M NaOH (sodium hydroxide, Merck Millipore, Darmstadt, Germany), 1 M HEPES buffer (4-(2-Hydroxyethyl)piperazine-1-ethanesulfonic acid dissolved in UPW, Sigma-Aldrich, Schnelldorf, Germany), and 10× DMEM (10× concentrated Dulbecco’s Modified Eagle’s Medium, Biochrom, Berlin, Germany) at a ratio of 1:1:2. Solution A was mixed with four parts of 0.5% collagen solution (Collagen G1 from bovine calf skin, Matrix BioScience, Mörlenbach, Germany) to obtain solution B. To avoid early gelation of the neutralized collagen solution, all chemicals were refrigerated (4–8 °C). The surface-functionalized scaffolds were placed in a 48-well plate, dripped by the collagen solution, and incubated over night at 37 °C to initiate and complete the collagen fibrillogenesis. After gelation, samples were completely dried under the hood.

#### 4.1.4. Crosslinking

For further stabilization of the collagen coating, samples were additionally crosslinked (cl). According to Powell et al. [57], a 50 mM MES (4-Morpholineethanesulfonic) acid solution in 40 vol % ethanol (Ethanol EMSURE^®^, Merck Millipore, Darmstadt, Germany) was prepared using MES hydrate (Sigma-Aldrich, Schnelldorf, Germany). 60 mM EDC (*N*-3-Dimethylaminopropyl)-*N*′ethylcarbodiimide, Sigma-Aldrich, Schnelldorf, Germany) and 60 mM NHS (*N*-Hydroxysuccinimide, Sigma-Aldrich, Schnelldorf, Germany) were dissolved in the MES buffer solution. Collagen-coated bioactive glass-based scaffolds were immersed in the crosslinking solution for 4 h at RT. Afterwards, samples were removed and washed in 0.1 M Na_2_HPO_4_ (disodium hydrogen phosphate, Sigma-Aldrich, Schnelldorf, Germany) for 2 h to hydrolyze remaining *O*-acylisourea of the carbodiimide [58,59]. Subsequently, scaffolds were washed in UPW and left to dry in air. Uncrosslinked samples are labeled as “uc”.

### 4.2. Evaluation of Bioactivity

For the assessment of acellular bioactivity in vitro, uncoated and collagen-coated samples were immersed in simulated body fluid (SBF), applying the well-known protocol introduced by Kokubo and Takadama [60]. Samples were put in closable containers of polypropylene, immersed in 50 mL of SBF, and placed in an orbital shaker (90 rpm, 37 °C). After different time points, samples were removed, washed in UPW, and left for drying. The formation of hydroxyapatite on the surface of the scaffolds, as a marker of bioactivity, was evaluated by scanning electron microscopy (SEM) and Fourier transform infrared spectroscopy (FTIR).

### 4.3. Release Behavior

The release behavior of collagen-coated BG-based scaffolds with and without crosslinks was evaluated in different media. Samples were immersed in 5 mL of PBS or SBF, respectively, and placed in an orbital shaker at 37 °C and 90 rpm. After different time points, the solution was completely removed and refilled by fresh solution. The released collagen was determined colorimetrically based on a modification of Lowry’s method [61,62]. For this analysis, 250 µL of protein solution was incubated with 250 µL of Lowry reagent solution (Sigma-Aldrich, Schnelldorf, Germany) for 20 min at RT. Afterwards, 125 µL of 0.3 N Folin & Ciocalteu’s phenol reagent (Sigma-Aldrich, Schnelldorf, Germany) was added and incubated for an additional 30 min to allow the color to develop. The solution was irradiated by visible light at 595 nm using a single beam spectrometer (Specord 40, Analytik Jena, Jena, Germany) and absorbance values were recorded. The collagen concentration was determined from a standard calibration curve.

### 4.4. Cell Studies

#### 4.4.1. Cell Culture and Seeding

For static cell culture experiments, a human osteosarcoma cell line (MG-63, Sigma-Aldrich, Schnelldorf, Germany) was used. MG-63 are adherent cells received from a malignant bone tumor and are commonly used as osteoblastic model cells [63]. Cells were cultured in low glucose Dulbecco’s Modified Eagle Medium (DMEM, Thermo Fisher Scientific, Schwerte, Germany) supplemented with 10 vol % of fetal bovine serum (FBS, Sigma-Aldrich, Schnelldorf, Germany) and 1 vol % of penicillin–streptomycin (PenStrep, Thermo Fisher Scientific, Schwerte, Germany) in an incubator at 37 °C with 5% of CO_2_ and 95% humidity. Cells were collected by trypsinization at confluency between 80% and 100%. Scaffolds were seeded with 600,000 cells/mL. The medium was changed twice a week.

#### 4.4.2. Cell Viability and Relative Proliferation

Cell viability was measured after 7, 14, and 21 days as an indirect measurement of the viable cell number using a cell counting kit (Cell Counting Kit-8, Sigma-Aldrich). Samples were washed with PBS and incubated with 1 vol % WST-8 in DMEM. After 4 h of incubation, absorbance was measured at 450 nm (PHOmo, Autobio Labtec Instruments, Zhengzhou, China).

The relative proliferation was determined by an enzyme-linked immunosorbent assay (ELISA) using a cell proliferation kit (Cell Proliferation ELISA, Bromodeoxyuridine (BrdU), Roche, Mannheim, Germany). Shortly summarized, samples were incubated with a BrdU labeling solution for 2 h at 37 °C. During incubation, BrdU is incorporated into the DNA of proliferating cells. Afterwards, cells were fixed and incubated with an antibody. As the antibody was detected immunohistochemically, the reaction was stopped by addition of 1 M H_2_SO_4_ (Sulfuric acid, EMSURE^®^ ISO, Merck Millipore, Darmstadt, Germany) and absorbance was measured at 450 nm.

#### 4.4.3. Cell Morphology

The morphology of seeded MG-63 cells on top of uncoated and collagen-coated bioactive glass-based scaffolds was investigated by SEM. After incubation, samples were washed in PBS and fixed with fixative I and II (Table 3) for 1 h, respectively. After fixation, samples were dehydrated in a graded ethanol series (30, 50, 70, 80, 90, 95, and 99.8 vol %) and dried in a critical point dryer (EM EPD300, Leica, Germany).

### 4.5. Methods

#### 4.5.1. Scanning Electron Microscopy (SEM)

For the observation by SEM, an Ultra plus scanning electron microscope (Auriga, Zeiss, Jena, Germany) was used. Samples were fixed with conductive silver, sputtered with gold (Quorum Q150T S, Quorum Technology, Darmstadt, Germany), and examined with 2 kV at a working distance of ~5 mm.

#### 4.5.2. X-ray Photoelectron Spectroscopy (XPS)

To confirm the presence of APTS molecules on the bioactive glass surface, X-ray photoelectron spectroscopy (XPS) was applied. The measurement was carried out by a multi-technique XPS (Phi-5600, Al-Kα radiation).

#### 4.5.3. Fourier Transform Infrared Spectroscopy (FTIR)

For chemical analysis, FTIR was used. 1 wt % of the sample to be analyzed was mixed with KBr (potassium bromide for IR spectroscopy Uvasol^®^, Merck Millipore, Darmstadt, Germany) and pressed into a pellet. The spectra were recorded in absorbance mode (Nicolet 6700 FTIR spectrometer, Thermo Scientific, Waltham, MA, USA) and collected between 4000 and 400 cm^−1^. Pure KBr was used to correct the background noise.

#### 4.5.4. Thermogravimetric Analysis (TGA)

In order to quantify the amount of collagen on the surface of coated scaffolds, thermogravimetric analysis was carried out. Heating was performed in air with a heating rate of 5 K/min (TGA, STA 449 F3 Jupiter, Netzsch, Selb, Germany).

#### 4.5.5. Mechanical Characterization

The compressive strength of uncoated and collagen-coated scaffolds (with and without crosslinks) was evaluated by uniaxial compression tests. Samples were compressed with a crosshead velocity of 1 mm/min, a preload of 0.1 N, and a maximum applied force of 50 N (Z050, Zwick, Ulm, Germany).

### 4.6. Statistical Analysis

Statistic evaluation of the results of cell studies was performed by one-way ANOVA (Analysis of Variance, Origin 8.6) with significance levels at *p* < 0.05, ** *p* < 0.01, and *** *p* < 0.001, using Bonferroni’s post-hoc test.

## 5. Conclusions

It can be concluded that collagen is an advantageous coating material for bioactive glass-based scaffolds. Relatively thick collagen layers (of a few micrometers) were applied on scaffolds without affecting the scaffold macroporosity. The compressive strength of BG-based scaffolds was increased by the presence of collagen, in particular by a factor of five for crosslinked collagen-coated scaffolds. Cell culture studies (MG-63 cells) demonstrated cell viability and suitable cell attachment and proliferation. Comparative studies considering alternative natural biopolymers as coatings of BG scaffolds are required to fully assess the relative advantages of collagen in this application. The results of this study provide a well-founded basis for future investigations in this field, in particular for further in vivo characterization of the produced composite scaffolds.

## Figures and Tables

**Table 1:** X-ray Photoelectron Spectroscopy (XPS) analysis showing the atomic concentration for each element present on the surface before and after the functionalization process.

	Atomic Concentration				
	N1s	Si2p	Ca2p	Na1s	P2p
**Before**	0.01	6.58	5.83	24.68	4.25
**After**	3.29	11.36	3.07	9.42	2.54

**Table 2:** Selected scaffolds. Collagen release was determined depending on the cleaning and functionalization process (- not applied, + applied).

Sample	Cleaning	Functionalization	Collagen	Crosslinking
**●** **(A)**	-	-	+	-
**◆** **(B)**	+	-	+	-
**■** **(C)**	-	+	+	-
**▲** **(D)**	+	+	+	-

**Table 3:** Composition of fixative I and II.

Reactant	Fixative I	Fixative II
Sodium cacodylate trihydrate (Sigma-Aldrich, Germany)	0.2 M	0.2 M
Glutaraldehyde (AppliChem, Germany)	0.1 wt %	0.3 wt %
Paraformaldehyde (Sigma-Aldrich, Germany)	2 wt %	3 wt %
Sucrose (Sigma-Aldrich, Germany)	5 wt %	-
